# Current management of primary mitochondrial disorders in EU countries: the European Reference Networks survey

**DOI:** 10.1007/s00415-023-12017-1

**Published:** 2023-10-13

**Authors:** Michelangelo Mancuso, Piervito Lopriore, Costanza Lamperti, Thomas Klopstock, Shamima Rahman, Laura Licchetta, Cornelia Kornblum, Saskia B. Wortmann, Hélène Dollfus, Maria T. Papadopoulou, Alexis Arzimanoglou, Maurizio Scarpa, Holm Graessner, Teresinha Evangelista

**Affiliations:** 1 Department of Clinical and Experimental Medicine, Neurological Institute, University of Pisa, Pisa, Italy; 2 Unit of Medical Genetics and Neurogenetics, Fondazione IRCCS Istituto Neurologico Carlo Besta, Milan, Italy; 3 Friedrich-Baur-Institute, Department of Neurology, LMU University Hospital, Ludwig-Maximilians-Universität München, Ziemssenstraße 1a, 80336 Munich, Germany; 4 German Center for Neurodegenerative Diseases (DZNE), Munich, Germany; 5 Munich Cluster for Systems Neurology (SyNergy), Munich, Germany; 6 UCL Great Ormond Street Institute of Child Health, London, UK; 7 IRCCS Istituto delle Scienze Neurologiche di Bologna, European Reference Network for Rare and Complex Epilepsies (EpiCARE), Bologna, Italy; 8 Department of Neurology, University Hospital Bonn, 53127 Bonn, Germany; 9 University Children’s Hospital, Paracelsus Medical University (PMU), Salzburg, Austria; 10 Centre de Référence pour les affections rares en génétique ophtalmologique (CARGO), Hôpitaux Universitaires de Strasbourg, ERN-EYE coordination, UMRS_1112 Institut de Génétique Médicale d’AlsaceI, GMA 67000 Strasbourg, France; 11 Paediatric Epilepsy Department, ERN EpiCARE, University Hospitals of Lyon (HCL), Lyon, France; 12 Neurology Department, Epilepsy unit, ERN EpiCARE coordination, Hospital San Juan de Dios, Barcelona, Spain; 13 Regionale Coordinating center for rare Diseases, MetabERN coordination, University Hospital Udine, Udine, Italy; 14 Institute for Medical Genetics and Applied Genomics, Centre for Rare Diseases, ERN RND coordination, University Hospital Tübingen, Tübingen, Germany; 15 Institute of Myology, EURO-NMD coordination, Pitié-Salpêtrière Hospital, APHP Sorbonne University, Paris, France

**Keywords:** Europe, European Reference Networks, Mitochondrial diseases, Rare diseases, Survey

## Abstract

**Background and purpose:**

Primary mitochondrial diseases (PMDs) are rare diseases for which diagnosis is challenging, and management and training programs are not well defined in Europe. To capture and assess care needs, five different European Reference Networks have conducted an exploratory survey.

**Methods:**

The survey covering multiple topics relating to PMDs was sent to all ERNs healthcare providers (HCPs) in Europe.

**Results:**

We have collected answers from 220 members based in 24/27 European member states and seven non-European member states. Even though most of the responders are aware of neurogenetic diseases, difficulties arise in the ability to deliver comprehensive genetic testing. While single gene analysis is widely available in Europe, whole exome and genome sequencing are not easily accessible, with considerable variation between countries and average waiting time for results frequently above 6 months. Only 12.7% of responders were happy with the ICD-10 codes for classifying patients with PMDs discharged from the hospital, and more than 70% of them consider that PMDs deserve specific ICD codes to improve clinical management, including tailored healthcare, and for reimbursement reasons. Finally, 90% of responders declared that there is a need for further education and training in these diseases.

**Conclusions:**

This survey provides information on the current difficulties in the care of PMDs in Europe. We believe that the results of this survey are important to help rare disease stakeholders in European countries identify key care and research priorities.

**Supplementary Information:**

The online version contains supplementary material available at 10.1007/s00415-023-12017-1.

## Introduction

Primary mitochondrial diseases (PMDs) are a heterogeneous group of genetic disorders that affect the structure or function of the mitochondrial oxidative phosphorylation (OXPHOS) system and are a consequence of pathogenic variants in either nuclear DNA (nDNA) or mitochondrial DNA (mtDNA) [1]. PMDs comprise one of the most common groups of inherited metabolic disorders, with an estimated prevalence of approximately 1:5000 adults and 5–15 cases per 10,000 children [2]. This is one of the main reasons why physicians involved in the rare diseases field must be aware of and prepared to manage these diseases. Therefore, there is a growing worldwide attention on PMDs, with multiple areas of medical sciences and stakeholders sharing this interest.

Due to the significant clinical and genetic heterogeneity, both for the many genes involved (genetic heterogeneity) and the great variety of mutation types in a single gene (allelic heterogeneity), PMDs are often challenging to diagnose [3]. The diagnostic process may take years [4], require several specialists, and need multiple medical investigations. However, genetic diagnosis is increasingly recognized as mandatory for PMDs since it allows proper counselling, family planning, and access to multi organ surveillance and therapies or clinical trials.

Genetic diagnostic services are already under considerable pressure to integrate new technologies and discoveries and to ensure equal access and rapid turnaround in order to avoid treatment and management delays. Moreover, evidence suggests that PMDs present a large economic burden on the healthcare system. In the United States, the average cost per month for treatment of PMDs has been estimated to be between $3100 and $4829, considerably higher than the average healthcare costs in the population [5]. A similarly high economic burden was recently published in Canada, where the mitochondrial disease population experienced a high need for health care and incurred high costs (mean = CAD$24,023 in 12 months before first hospitalization) within the single-payer Ontario health care system [6].

The economic burden of PMDs in the European health systems is mostly unknown. Moreover, the quality of the awareness and training about PMDs among medical school and residency programs is incompletely understood at the European level.

In March 2017, 24 virtual European Reference Networks (ERNs) were launched involving healthcare providers (HCPs) across Europe. They aim to tackle complex or rare diseases and conditions that require highly specialized treatment and concentration of knowledge and resources. The 24 ERNs involve 25 European countries including Norway (and until brexit UK), more than 300 hospitals with over 900 healthcare units and covering all major rare disease groups (https://health.ec.europa.eu/european-reference-networks/networks_en). Although different ERNs cover different disease areas, some disease groups have overlapping features and may be in the remit of several ERNs. Based on these commonalities, the ERNs EURO-NMD (for rare neuromuscular diseases), RND (for rare neurological diseases), EpiCare, (for rare epilepsies) MetabERN (rare metabolic diseases) and ERN EYE (rare ophthalmological diseases) came together to establish an inter-ERNs working group that aims to develop common work around care, education and research on PMDs, called the Mito InterERNs. The group consists of nine experts in mitochondrial diseases plus two patients’ representatives (from Mitocon Italy and Lily Foundation UK).

The aim of this work is to gather information on different aspects of PMDs, as understood by the European HCPs affiliated to the 24 different ERNs. Topics of interest in this survey were: (i) general interest in PMDs; (ii) ICD codes for PMDs; (iii) provision of muscle biopsy and genetic services in Europe; (iv) presymptomatic, prenatal and preimplantation genetic diagnosis for PMDs; (v) management of PMDs and (vi) education on PMDs.

## Methods

The current project is a cross-sectional survey focused on members of five ERNs, who deal with both adult and paediatric patients affected by PMDs and whose clinical practice is performed in Europe. The Mito InterERNs group met online three times and all questions were designed and approved by the Mito InterERNs group delegates (see supplementary file 1), taking into account the following elements at the national level: awareness of PMDs, national policies, reimbursement codes, access to different diagnostic tests, education needs and training. The survey was tested within the group and then distributed to all European HCPs affiliated to the 24 different ERNs through the official ERNs channels (including the official ERNs mailing lists, newsletters, social media accounts) and it was conducted online, with 3 reminders, between November 3rd, 2022, and April 31st, 2023. Responses were collected through the Google forms platform and then analyzed anonymously.

## Results

**Fig. 1:**
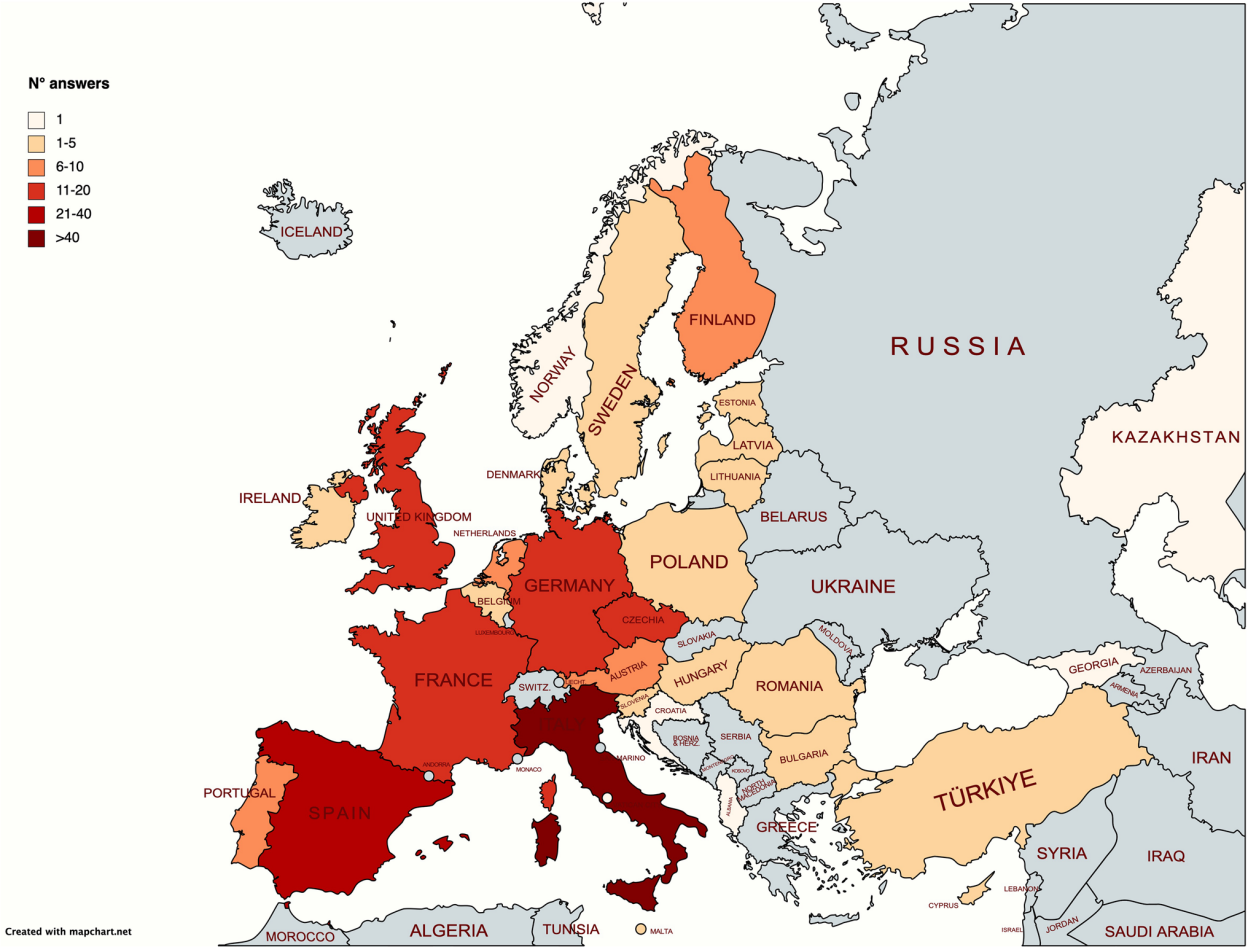
European countries affiliated to the ERNs where we have obtained responses to the survey

**Fig. 2:**
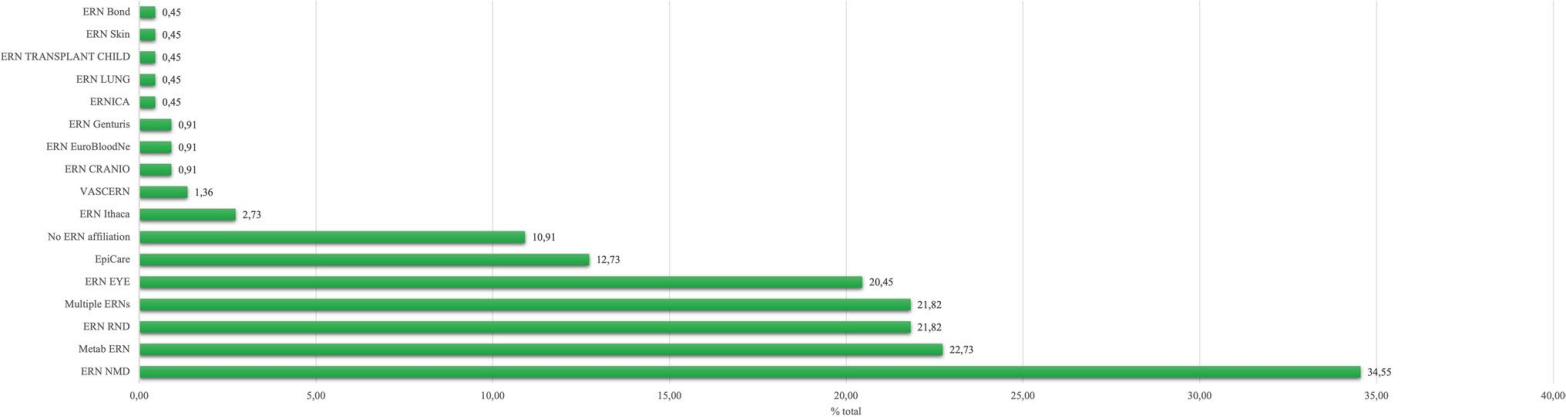
Responses obtained from different ERNs

The full list of queries and results are reported in supplementary files 1 and 2, respectively. A total of 220 responders completed the survey (64% female), representative of 24/27 European member states plus Albania, Georgia, Kazakhstan, Norway, Turkey, United Kingdom, Vatican City (Fig. 1) and of 15/24 different ERNs (Fig. 2). Of those, 40% were neurologists, 19% paediatricians, 9% geneticists, and 19% other specialists affiliated to different ERN HCPs.

### General aspects

Almost all participants (99.5%) were aware of PMDs and thought (93.2%) that they represent an important group of diseases. Most of the respondents (91.8%) follow patients with PMDs, mainly mitochondrial myopathy (67.7%), Leber hereditary optic neuropathy (LHON) and autosomal dominant optic atrophy (ADOA) (53.2%), mitochondrial encephalomyopathy with lactic acidosis and stroke-like episodes (MELAS) (67.3%), myoclonic epilepsy ragged red fibres (MERRF) (48.6%) Kearns-Sayre syndrome (60.9%) and Leigh syndrome (56.4%). While all the responders agreed that family history, including matrilineal inheritance, is an important finding in the diagnostic flowchart, in almost 17% of cases, this information is either not collected or incompletely collected, which could contribute to incorrect or delayed diagnosis. An important message emerging from the survey is that only 12.7% were happy with the ICD-10 codes for classifying PMDs discharged from the hospitals, while more than 50% were not, and more than 70% of responders thought that PMDs deserve specific ICD-10 codes for reimbursement reasons or for better attention and tailored healthcare.

In a patient with a possible PMD, almost 86% of the respondents directly request appropriate genetic testing, while 30.5% would refer the patient to a subspecialist for diagnostic testing.

Almost 26% of responders declared that presymptomatic screening is not possible or they are not aware of it as a possibility. Different specialists with expertise in PMDs are allowed to request genetic testing for 66% of responders; however, presymptomatic genetic testing is preceded by a medical genetic counselling consultation in more than 80% of cases. Finally, almost all responders who are aware of prenatal diagnosis testing (83%) declare that this needs to be preceded by counselling by a clinical geneticist considering the complexity of inheritance of PMDs. We should be aware of the risks of performing presymptomatic -and more rarely prenatal genetic testing by non-geneticists without input from specialist clinical geneticists; however, in most cases (82 and 84.7%, respectively), genetic testing is preceded by a medical genetic counselling consultation.

### Availability of genetics tests

**Fig. 3:**
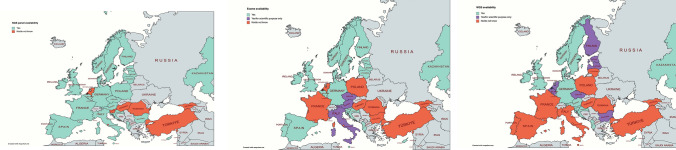
Diagnostic availability based on survey responders. It was considered Yes or Yes/for Scientific purpose only if “Yes” or “Yes” plus “for scientific purpose only” were > 66.6% of total answers, otherwise, it was considered as no/do not know

Even though most of the responders were aware of PMDs, the ability to perform complete genetic diagnostic testing varies greatly between different HCPs. While single gene analysis or next generation sequencing (NGS) panels are widely available (85.9% and 77.7%, respectively) in the different European countries, whole exome (WES) and genome (WGS) sequencing are not easily accessible for many of the responders (see Fig. 3) where the European situation appears to vary from country to country. These discrepancies between countries and the completeness of the acquired data cannot be fully evaluated owing to the limited number of answers we received in this survey.

Another important issue is the latency between the test request and the availability of a genetic result. In more than 40% of cases, it takes more than 3 months for single gene analysis and almost 60% for NGS panels to be reported; in more than 30% of cases analyzed by WES, it takes more than 6 months (40% in the case of WGS).

### Prescription of specific mitochondrial compounds on market

60% of the responders prescribe a mitochondrial “cocktail” (a mixture of vitamins and cofactors with varying effects on the mitochondrial respiratory chain) to patients affected by PMDs, while 40% do not. Coenzyme Q_10_ (and its analogues), riboflavin and l-carnitine are the most prescribed supplements (85%, 64.7% and 60.5%, respectively). Discrepancy in terms of reimbursement by the different health systems is evident: 40% of responders declare that mitochondrial supplements are reimbursed, while this is not the case for 39.1% of responders.

### Education

Nearly 91% of responders declared that there is a need for education and training in PMDs. More ERN educational activities are encouraged by 90% of responders.

## Discussion

PMDs comprise one of the most common rare diseases; tens of thousands of patients in Europe are affected at any age [7]. As for other rare diseases, management of patients with PMDs presents a significant challenge [8, 9] to health policy makers, health care providers, patients, and society. Contributory factors include gaps in knowledge, lack of awareness, and difficulties in genetic testing and management, with high associated costs.

This survey has collated information on different health-related activities for PMDs, which may help to improve and homogenize health care service to the rare disease community.

We are aware that our survey tool has some limitations. Firstly, the survey did not collect sufficient responses from paediatricians, with only 16.4% of responders being involved in the care of children. Secondly, although a large proportion of European countries were included in the study, for some of them we obtained a very low number of responders (Fig. 1), and this might have biased the results, at least for those countries. Thirdly, several upcoming European countries were either not reached by our survey or have only answered minimally; this could be partially explained by the fact that these countries are underrepresented within the ERNs, and more data are needed. Finally, the results were based on the responses from a single physician for each HCP. This might also have biased the results of care needs in some European countries. However, more than 90% of respondents were affiliated to one (or multiple) ERNs, and we assume they were likely to be well informed about the PMDs in their respective country. It would be important in the future to both replicate and expand our data to have more insights in the field of PMDs.

Even though absolute conclusions cannot be reached, several messages arise from this survey.

1. Despite the strong epidemiological impact of PMDs and the important costs related to them, education is still perceived as inadequate in most countries, as for other neurogenetic conditions [10]. Specific programs in PMDs in both medical school and residency curricula and in continuing medical education are strongly encouraged, as well as ERN initiatives providing more education about these diseases. Notably, several activities are already ongoing, including the MetabERN Academy, the education initiative of MetabERN which includes PMDs (https://metab.ern-net.eu/metabern-academy/) and the Moodle Platform under development by the ERN NMD with teaching courses consisting of webinars, bibliography suggestions and other teaching supportive tools (https://euronmd.lilicampus.com/login/index.php). Moreover, in collaboration with the European Academy of Neurology, a RD modular postgraduate curriculum is being developed, with comprehensive coverage of mitochondrial diseases. Finally, ERNs initiatives should also be developed in collaboration with existing mitochondrial scientific societies (i.e. the Mitochondrial Medicine Society or the European Society for Mitochondrial Research and Medicine), promoting shared initiatives, i.e. schools for the next generation of clinicians.
2. Information about prevalence and health care costs of mitochondrial diseases is not easy to collect, because ICD-10 codes are not well designed to properly classify rare diseases in general and PMDs specifically for reimbursement reasons or for better visibility and tailored healthcare. Moreover, within the present system, data on the economic burden of PMDs in Europe [6] are difficult to collect on a systematic level. This issue of accurate rare diseases codification in hospital information systems is currently being addressed by the growing use of the Orphanet nomenclature in European countries that allows a precise coding of all known rare diseases via so-called ORPHAcodes. (Ref: 10.1186/s13023-021-01763-y). In this context, the ERNs registries may represent a useful tool for data collection valuable for epidemiology and health care costs. Moreover, there is a process for requesting new/revised ICD codes (https://www.cms.gov/Medicare/Coding/ICD10/newrevisedcodes); and this will be discussed within the mito Inter-ERNs and the appropriate ERNs executive committee.
3. Despite many initiatives undertaken to facilitate the diagnosis and management of rare diseases including PMDs, in Europe, there is still much to be done to support these patients, including facilitating easier access to specific diagnostic gene testing, presymptomatic diagnosis, and newborn screening, also taking into account the challenges related to the complexity of inheritance patterns related to the PMDs.

We believe that this work may be of importance for all European stakeholders in rare diseases and PMDs to identify key priorities that should be addressed to do better in the near future. A deeper collaboration between all stakeholders in the arena (academia, researchers, physicians, politicians, patient advocacy groups and industries), is crucial to ensure that European patients receive the correct diagnosis faster wherever they live, the development of the best curriculum during both medical school and postgraduate training programmes and a continuous medical education program promoted by the different ERNs.

### Supplementary Information

Below is the link to the electronic supplementary material.

Supplementary file 1: The interERNs Survey queries. (PDF 278 KB)

Supplementary file 2: The interERNs Survey results. (PDF 3234 KB)

## Data Availability

The data that support this research are available from the corresponding author upon reasonable request.
