# Supplementary material for: Current management of primary mitochondrial disorders in EU countries: the European Reference Networks survey
Source: J Neurol. 2023 Oct 13;271(2):835–40. doi: 10.1007/s00415-023-12017-1 (PMC10828000; doi:10.1007/s00415-023-12017-1)
Supplement: Supplementary file 1 — The interERNs Survey queries. (PDF 278 KB) [file 415_2023_12017_MOESM1_ESM.pdf]

ERN Survey: How to approach and manage primary mitochondrial diseases (PMDs) in different EU countries. The ERNs survey

This survey is for MDs or residents affiliated to the ERN(s) and practicing in Europe (UK included)

*Required

1. Email *

____

2. Data protection. This survey is by invitation only. We will not share or make public information that can link you to individual responses presented in our final report. Do you agree to the above? *

Mark only one oval.

- ◯ Yes
- ◯ No

General information

3. GENDER *

Mark only one oval.

- ◯ MALE
- ◯ FEMALE
- ◯ Non-binary
- ◯ Prefer not to say
- ◯ Other: ______________________________

4. AGE RANGE (YEARS) *

Mark only one oval.

- UNDER 30
- 30-50
- MORE THAN 50

5. Current professional situation: *

Mark only one oval.

- NEUROLOGY RESIDENCY PROGRAMME
- NEUROLOGIST AFFILIATED TO HOSPITAL\UNIVERSITY
- NEUROLOGIST IN PRIVATE PRACTICE
- NEUROPEDIATRICIAN
- CLINICAL GENETIST
- OTHER MD SPECIALIST
- ePAG representative
- Other: ____________________

6. COUNTRY WHERE YOU PRACTICE (COUNTRY WHERE YOU LIVE IF YOU ARE A PAG member) *

Mark only one oval.

- Albania
- Andorra
- Armenia
- Austria
- Azerbaijan
- Belarus
- Belgium
- Bosnia Herzegovina
- Bulgaria
- Croatia
- Cyprus
- Czech Rep
- Denmark
- Estonia
- Finland
- France
- Georgia
- Germany
- Greece
- Hungary
- Iceland
- Ireland
- Italy
- Kazakistan
- Kosovo
- Latvia
- Liechtenstein
- Lithuania
- Luxembourg
- Malta
- Moldova

- Monaco
- Montenegro
- Netherlands
- North Macedonia
- Norway
- Poland
- Portugal
- Romania
- Russia
- San Marino
- Serbia
- Slovakia
- Slovenia
- Spain
- Sweden
- Switzerland
- Turkey
- Ukraine
- United Kingdom
- Vatican City

7. ERN(S) YOU ARE AFFILIATED (multiple choices allowed) *

Tick all that apply.

- [ ] ERN NMD
- [ ] ERN RND
- [ ] EPICare
- [ ] MetabERN
- [ ] ERN-EYE
- [ ] ERN BOND
- [ ] ERN CRANIO
- [ ] Endo-ERN
- [ ] ERKNet
- [ ] ERNICA
- [ ] ERN LUNG
- [ ] ERN Skin
- [ ] ERN EURACAN
- [ ] ERN EuroBloodNe
- [ ] ERN eUROGEN
- [ ] ERN GENTURIS
- [ ] ERN GUARD-HEART
- [ ] ERN ITHACA
- [ ] ERN PaedCan
- [ ] ERN RARE-LIVER
- [ ] ERN ReCONNET
- [ ] ERN RITA
- [ ] ERN TRANSPLANT-CHILD
- [ ] VASCERN
- [ ] NO ERN AFFILIATION

General interest in primary mitochondrial diseases (PMDs)

8. Do you think that PMDs have an important role in clinical practice? *

Mark only one oval.

- ( ) Yes
- ( ) No
- ( ) Do not know

9. Are you aware of PMDs? *

Mark only one oval.

- Yes
- No

10. Do you follow patients with PMDs? *

Mark only one oval.

- Yes
- No

11. If you follow patients with PMDs, which one of the listed groups are you familiar with (multiple choices allowed) *

Tick all that apply.

- [ ] Mitochondrial myopathy (including PEO)
- [ ] LHON and ADOA
- [ ] MELAS
- [ ] MERRF
- [ ] Kearns Sayre Syndrome
- [ ] Leigh syndrome
- [ ] Alpers disease
- [ ] Multisystem PMDs
- [ ] Others
- [ ] NONE

12. Do you think that a family history, including matrilinear inheritance, is usually collected? *

Mark only one oval.

- Yes
- No
- I do not know

13. Do you think that family history is an important finding in the diagnostic flowchart? *

Mark only one oval.

- Yes
- No
- Maybe

14. When you have a patient with a possible PMD, what is your attitude (multiple choices allowed): *

Tick all that apply.

- [ ] you send the patient to a subspecialist in the field
- [ ] you ask directly the appropriate gene testing
- [ ] you prescribe specific drugs and manage the follow up

PMDs codes

15. Are you happy with the ICD-10 codes for classifying PMDs discharged from your HCP? *

Mark only one oval.

- Yes
- No
- I do not know

16. Do you think PMDs deserve specific ICD codes for reimbursement reasons or for better attention and tailored healthcare for these patients *

Mark only one oval.

- Yes
- No
- I do not know

Analysis for PMDs diagnosis

17. Muscle biopsy available at your HCP *

Mark only one oval.

- Yes
- No
- I do not know

18. Single gene analysis available at your HCP *

Mark only one oval.

- YES
- NO
- I do not know

19. Single Gene analysis RESPONSE waiting time

Mark only one oval.

- < 3 months
- 3-6 months
- > 6 months
- I do not know

20. NGS Panels for PMDs available at your HCP *

Mark only one oval.

- Yes
- No
- I do not know

21. NGS Panels for PMDs RESPONSE waiting time

Mark only one oval.

- ◯ < 3 months
- ◯ 3-6 months
- ◯ > 6 months
- ◯ I do not know

22. Exome available at your HCP *

Mark only one oval.

- ◯ Yes
- ◯ No
- ◯ for scientific purpose only
- ◯ I do not know

23. Exome for PMDs RESPONSE waiting time *

Mark only one oval.

- ◯ <3 months
- ◯ 3-6 months
- ◯ > 6 months
- ◯ I do not know

24. Whole Genome (WGS) available at your HCP *

Mark only one oval.

- ◯ Yes
- ◯ No
- ◯ for scientific purpose only
- ◯ I do not know

25. WGS for PMDs RESPONSE waiting time

Mark only one oval.

- <3 months
- 3-6 months
- > 6 months
- I do not know

Presymptomatic diagnosis

If your answer to the first question is No/I do not know, please jump to next section

26. IS THE PRESYMPTOMATIC DIAGNOSIS SERVICE AVAILABLE IN YOUR COUNTRY? *

Mark only one oval.

- Yes
- No
- I do not know

27. IS THE PRESYMPTOMATIC DIAGNOSIS PRECEDED BY A MEDICAL GENETIC COUNSELING CONSULTATION?

Mark only one oval.

- Yes
- No
- I do not know

28. IS THE SPECIALIST WITH EXPERTISE IN PMDs ALLOWED TO PRESCRIBE GENETIC TESTING IN PRESYMPTOMATIC SUBJECTS?

Mark only one oval.

- Yes
- No
- I do not know

29. IS THE PRESYMPTOMATIC DIAGNOSIS SCREENING PERFORMED IN MINORS (<18-YRS)?

Mark only one oval.

- yes
- no
- ONLY IF THERE IS A TREATMENT ABLE TO CHANGE THE NATURAL HISTORY OF THE DISEASE
- I do not know

Prenatal diagnosis

If your answer to the first question is No/I do not know, please jump to next section

30. IS THE PRENATAL DIAGNOSIS SERVICE AVAILABLE IN YOUR COUNTRY? *

Mark only one oval.

- Yes
- No
- I do not know

31. IS THE PRENATAL DIAGNOSIS PRECEDED BY A MEDICAL GENETIC COUNSELING CONSULTATION?

Mark only one oval.

- Yes
- No
- I do not know

32. Which specialist is allowed to prescribe genetics test for prenatal diagnosis in your country (multiple choices allowed) *

Tick all that apply.

- [ ] Medical Genetist
- [ ] Gynecologist
- [ ] Pediatrician expert in PMDs
- [ ] Neurologist expert in PMDs
- [ ] Other MDs
- [ ] I do not know

Preimplantation genetic diagnosis (PGD)

33. IS PGD AVAILABLE IN YOUR COUNTRY? *

Mark only one oval.

- Yes
- No
- I do not know

34. Is there a law of the management of PGD in your country? *

Mark only one oval.

- Yes
- No
- I do not know

35. IS PGD PRECEDED BY A MEDICAL GENETIC COUNSELING CONSULTATION? *

Mark only one oval.

- Yes
- No
- I do not know

Management of PMDs

36. Do you prescribe the mitochondrial cocktail to your PMD patients *

Mark only one oval.

- Yes
- No
- I do not know

37. if you prescribe the mitochondrial cocktail, what do you usually consider (multiple choices allowed)

Tick all that apply.

- [ ] Coenzime Q10 and analogues
- [ ] Riboflavine
- [ ] carnitine
- [ ] lipoic acid
- [ ] vitamin E
- [ ] vitamin C
- [ ] Other
- [ ] None

38. Is the mitochondrial cocktail reimbursed by the health system of your Country? *

Mark only one oval.

- Yes
- No
- I do not know

EDUCATION ON MITOCHONDRIAL DISEASES

39. Is there a need for education and training in PMDs? *

Mark only one oval.

- Yes
- No
- I do not know

40. Is there a need for education and training in the residency programms in PMDs *

Mark only one oval.

- Yes
- No
- I do not know

41. Do you think ERNs must provide more education on PMDs through different initiatives *

Mark only one oval.

- Yes
- No
- I do not know

This content is neither created nor endorsed by Google.

Google Forms
