# Supplementary material for: Current management of primary mitochondrial disorders in EU countries: the European Reference Networks survey
Source: J Neurol. 2023 Oct 13;271(2):835–40. doi: 10.1007/s00415-023-12017-1 (PMC10828000; doi:10.1007/s00415-023-12017-1)

ERNs Survey on Primary mitochondrial diseases: Results

GENDER

220 responses

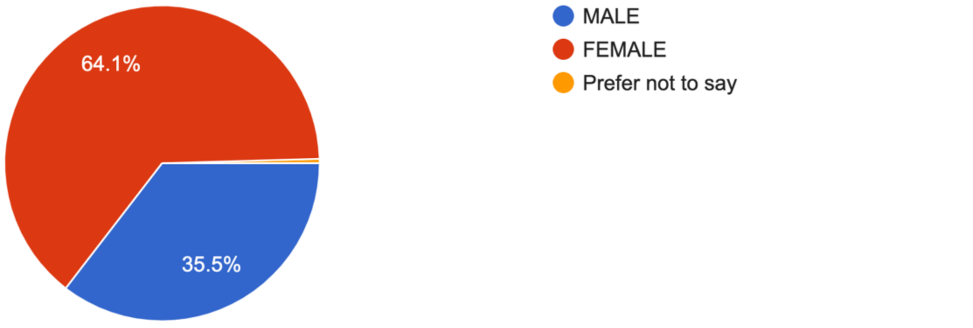

AGE RANGE (YEARS)

220 responses

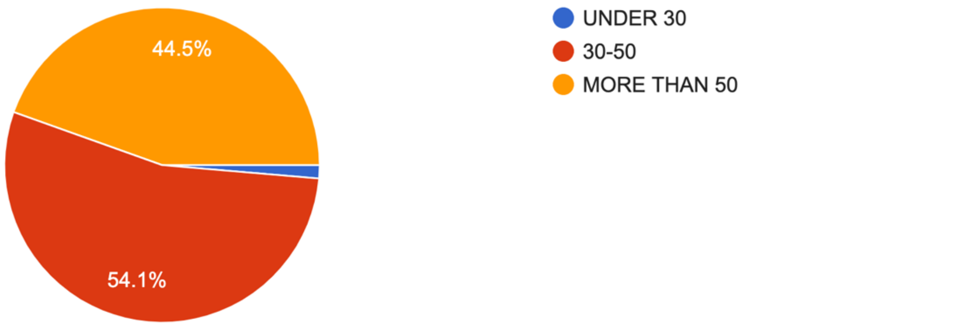

Current professional situation:

220 responses

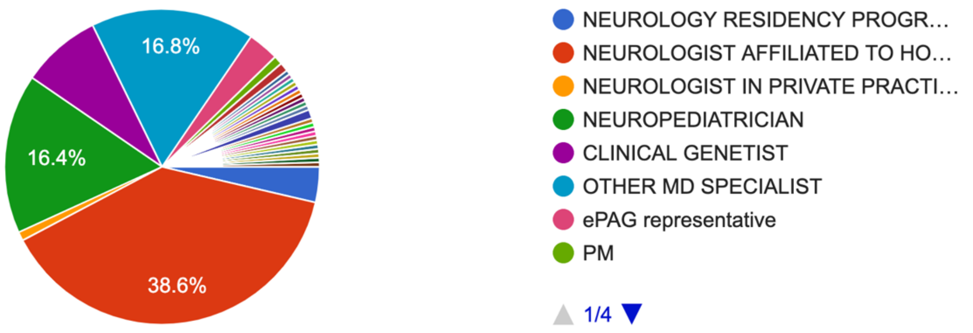

COUNTRY WHERE YOU PRACTICE (COUNTRY WHERE YOU LIVE IF YOU ARE A PAG member)

220 responses

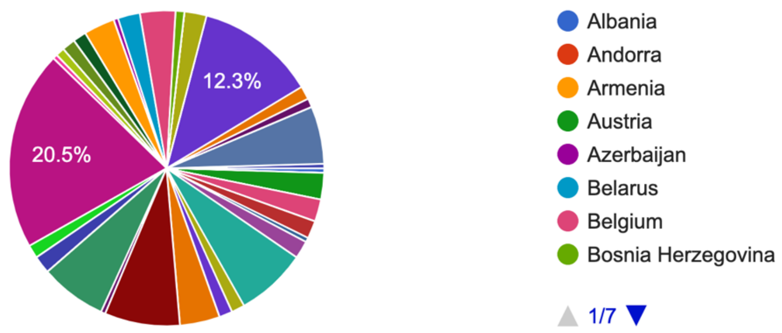

ERN(S) YOU ARE AFFILIATED (multiple choices allowed)

220 responses

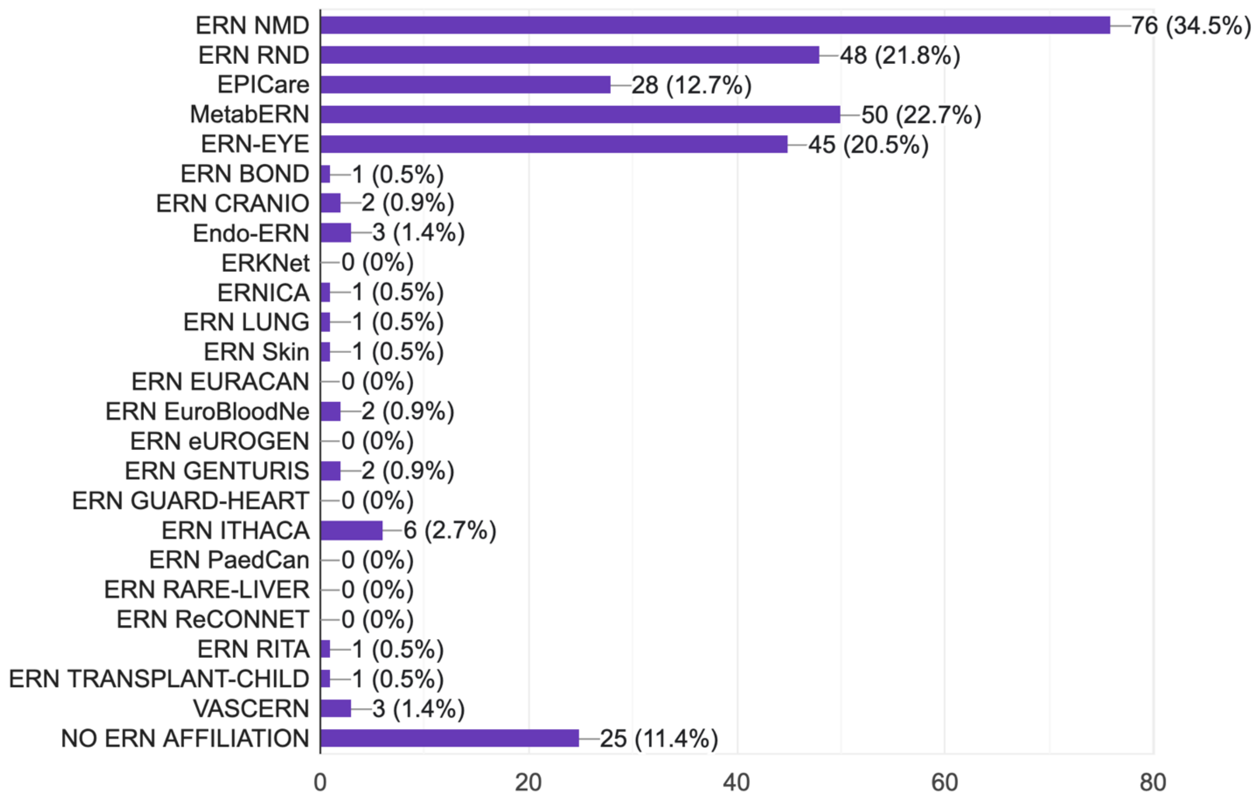

Do you think that PMDs have an important role in clinical practice?
220 responses

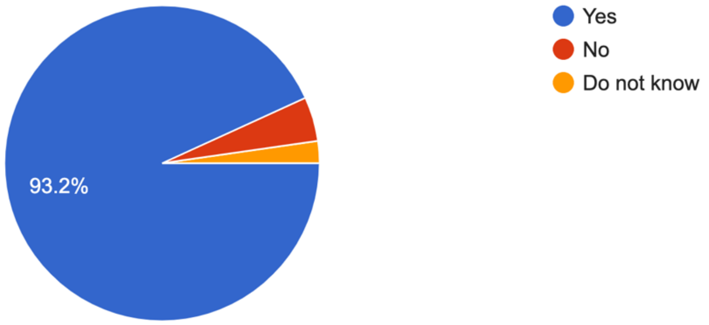

Are you aware of PMDs
220 responses

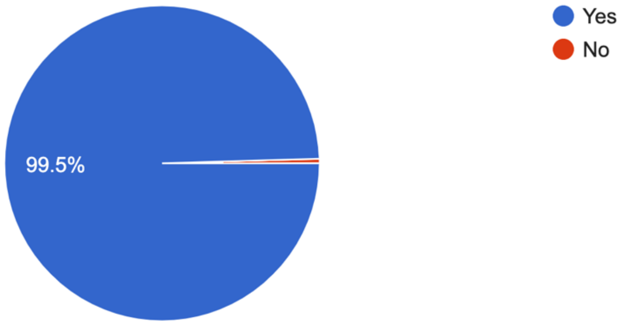

Do you follow patients with PMDs
220 responses

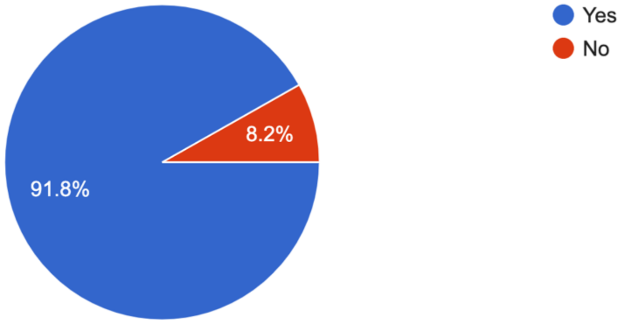

If you follow patients with PMDs, which one of the listed groups are you familiar with (multiple choices allowed)

220 responses

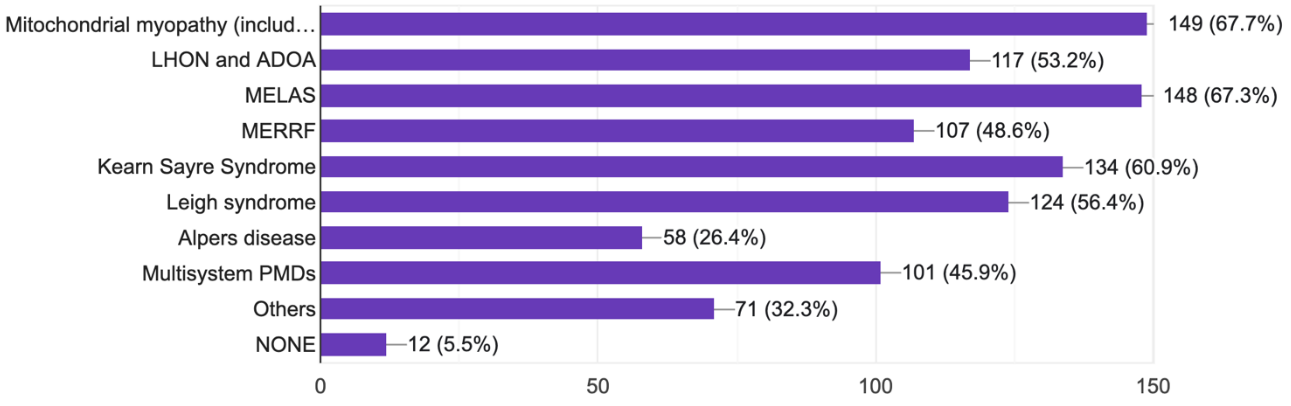

Do you think that a family history, including matrilinear inheritance, is usually collected?

220 responses

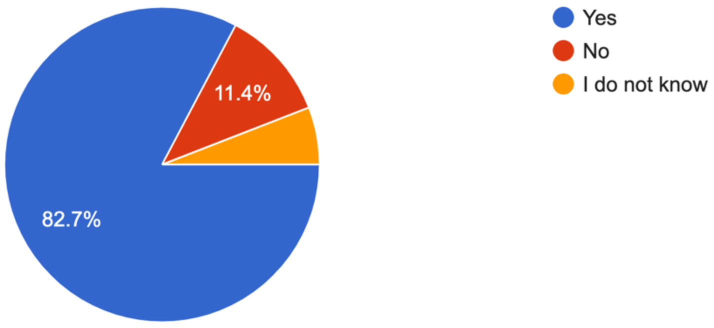

Do you think that family history is an important finding in the diagnostic flowchart?

220 responses

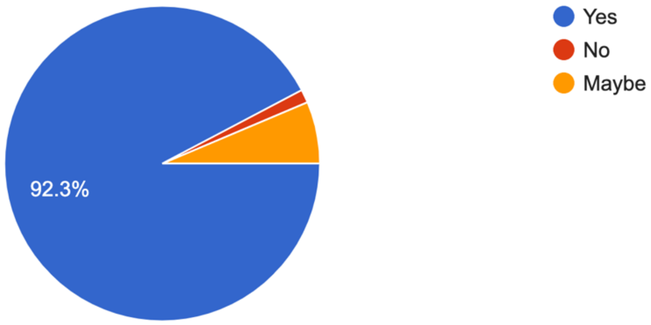

When do you have a patient with a possible PMD, what is your attitude (multiple choices allowed):
220 responses

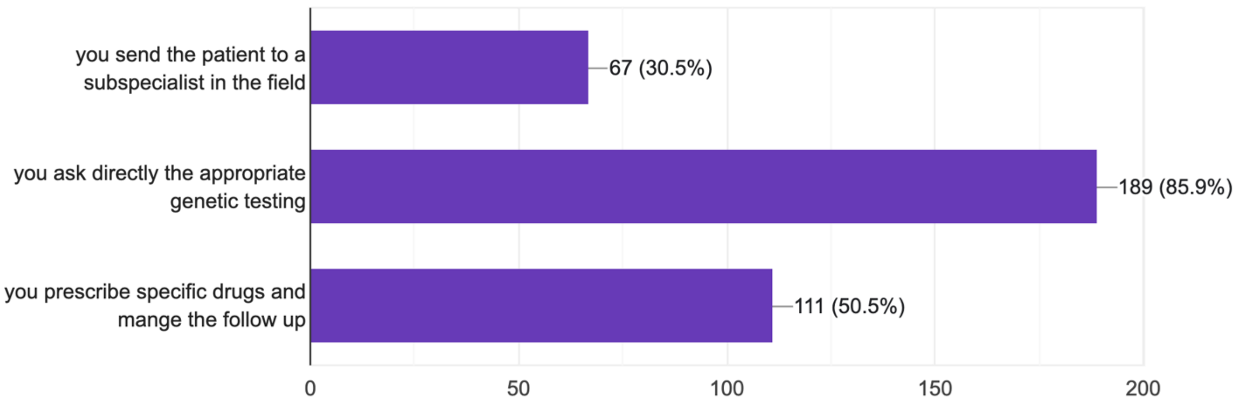

Are you happy with the ICD-10 codes for classifying PMDs discherged from your HCP?
220 responses

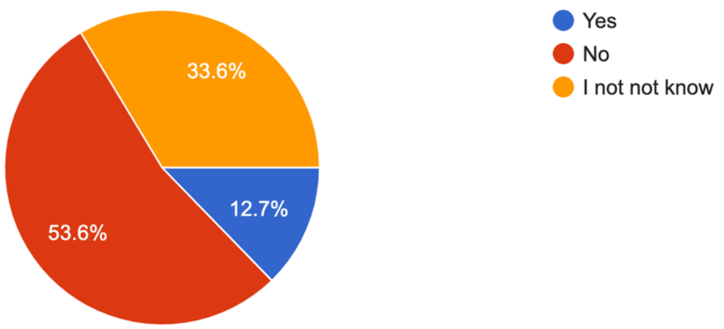

Do you think PMDs deserve specific ICD codes for reimbursement reasons or for better attention and tailored healthcare for these patients
220 responses

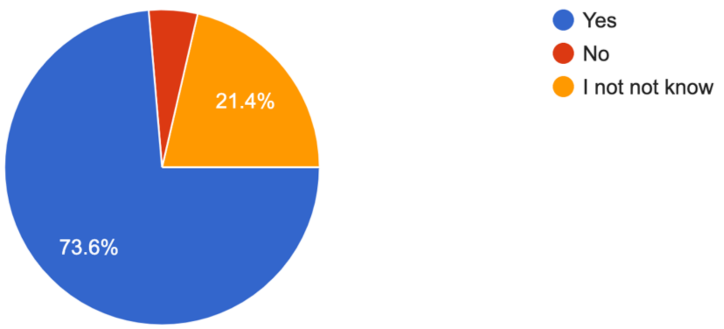

Muscle biopsy available at your HCP
220 responses

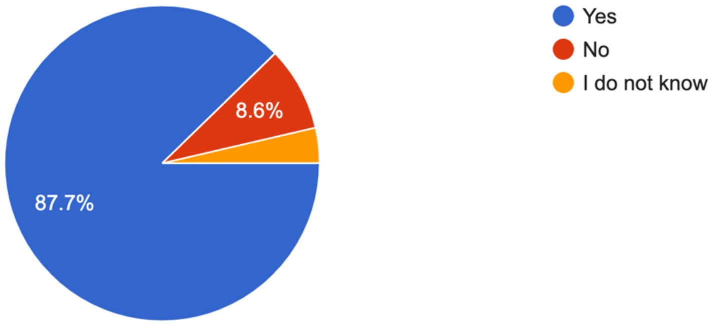

Single gene analysis available at your HCP
220 responses

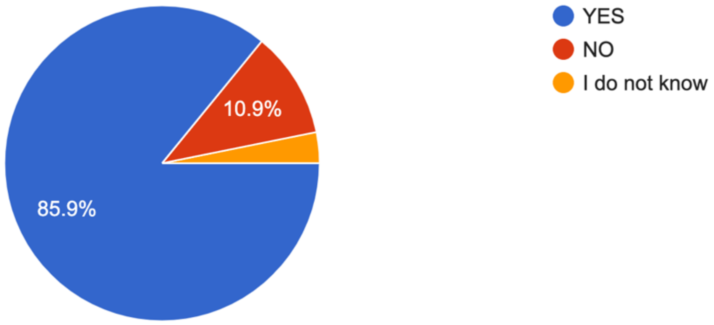

Single Gene analysis RESPONSE waiting time
220 responses

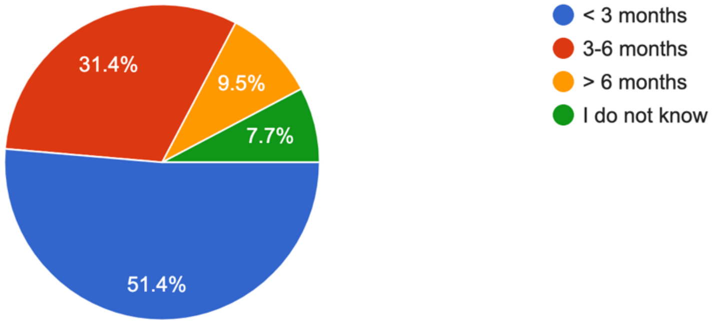

NGS Panels for PMDs available at your HCP

220 responses

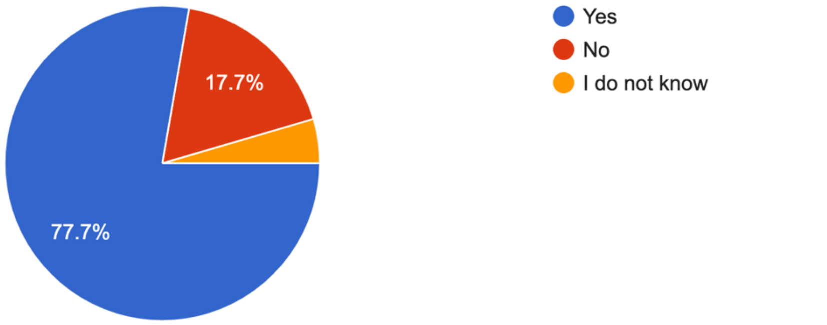

NGS Panels for PMDs RESPONSE waiting time

214 responses

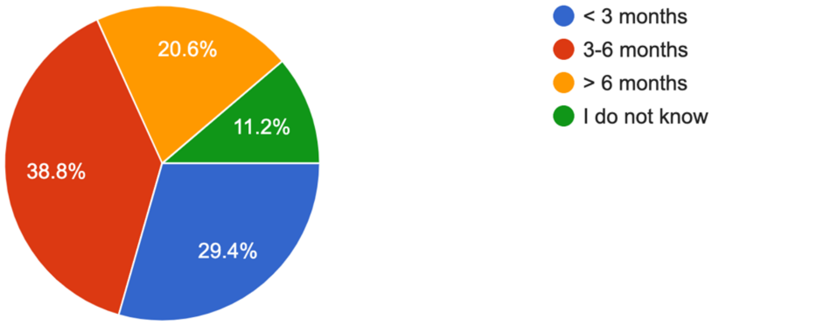

Exome available at your HCP

220 responses

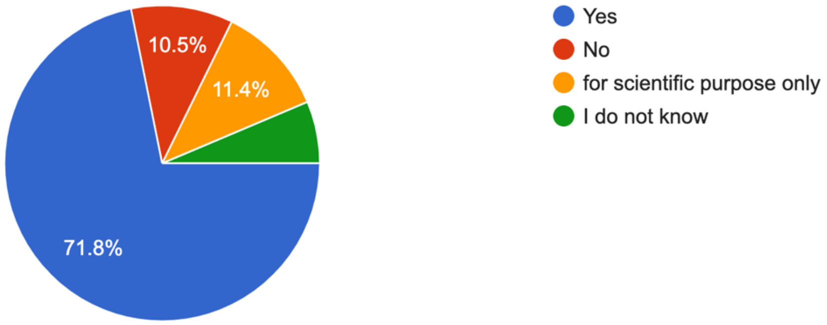

Exome for PMDs RESPONSE waiting time

220 responses

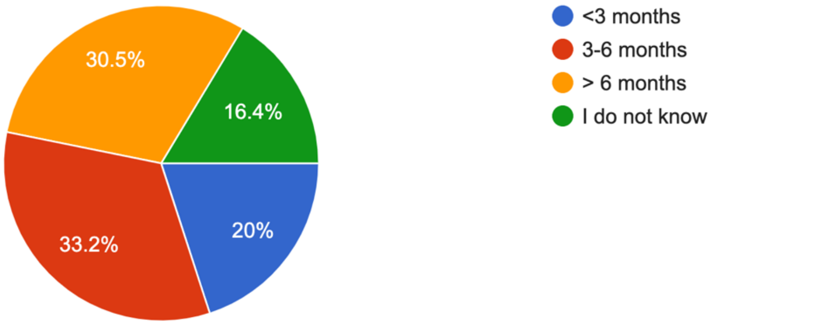

Whole Genome (WGS) available at your HCP

216 responses

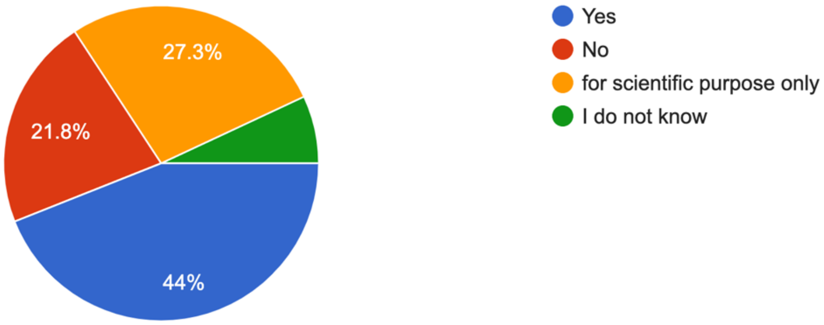

WGS for PMDs RESPONSE waiting time

205 responses

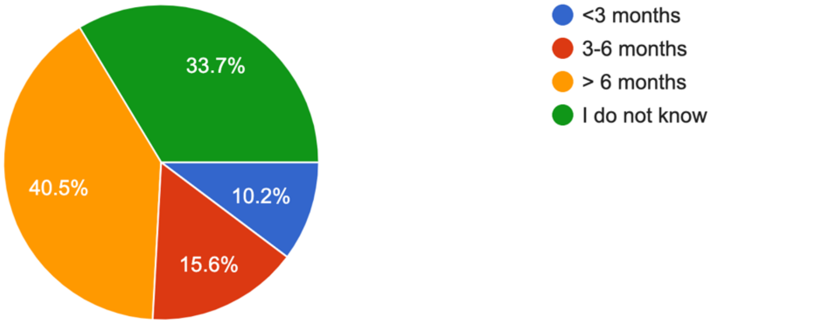

IS THE PRESYMPTOMATIC DIAGNOSIS SERVICE AVAILABLE IN YOUR COUNTRY?

220 responses

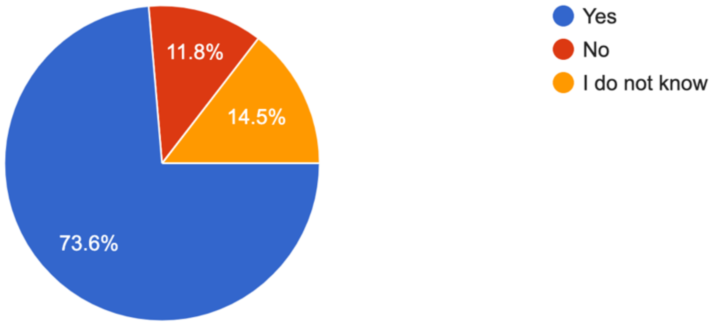

IS THE PRESYMPTOMATIC DIAGNOSIS PRECEDED BY A MEDICAL GENETIC COUNSELING CONSULTATION?

200 responses

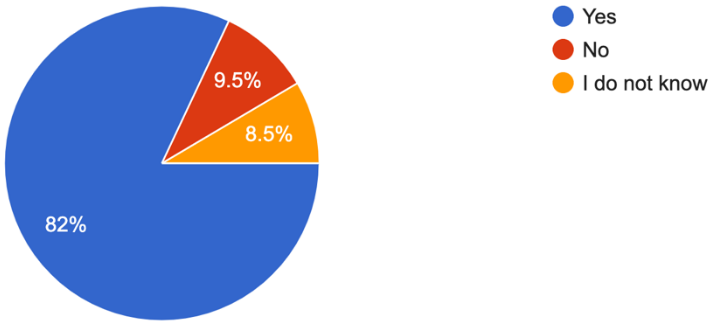

IS THE SPECIALIST WITH EXPERTISE IN PMDs ALLOWED TO PRESCRIBE GENETIC TESTING IN PRESYMPTOMATIC SUBJECTS?

200 responses

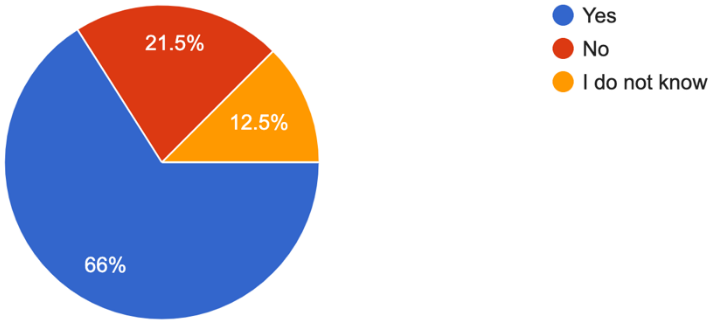

IS THE PRESYMPTOMATIC DIAGNOSIS SCREENING PERFORMED IN MINORS (<18-YRS)?

201 responses

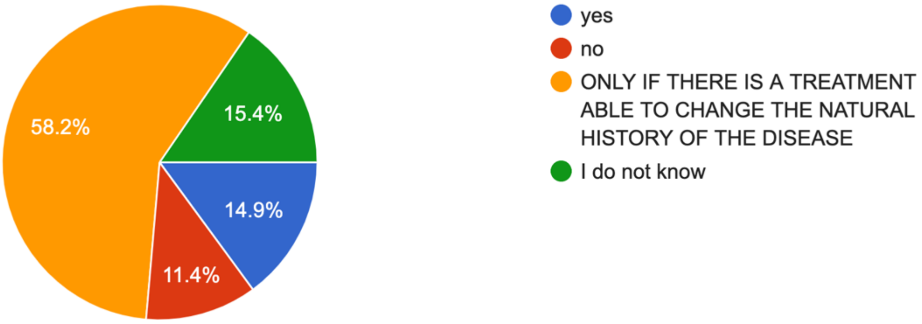

IS THE PRENATAL DIAGNOSIS SERVICE AVAILABLE IN YOUR COUNTRY?

220 responses

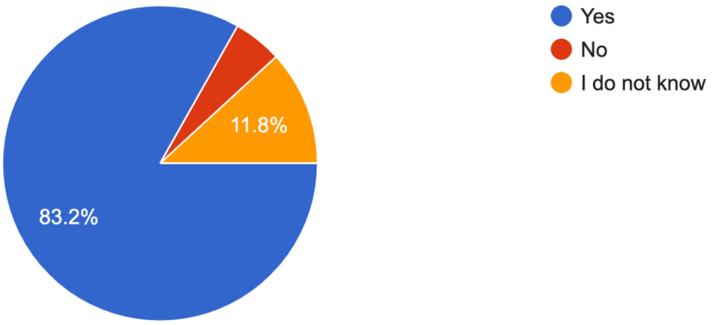

IS THE PRENATAL DIAGNOSIS PRECEDED BY A MEDICAL GENETIC COUNSELING CONSULTATION?

209 responses

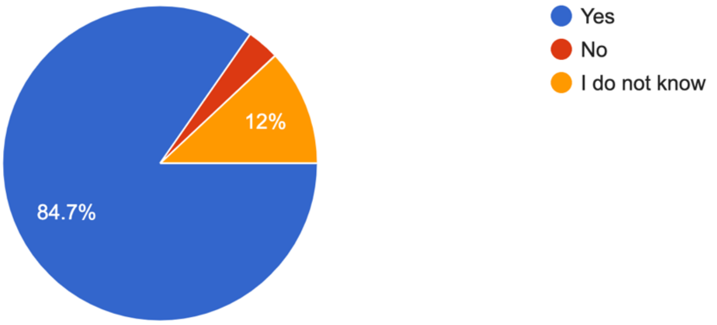

Which specialist is allowed to prescribe genetics test for prenatal diagnosis in your country (multiple choices allowed)

220 responses

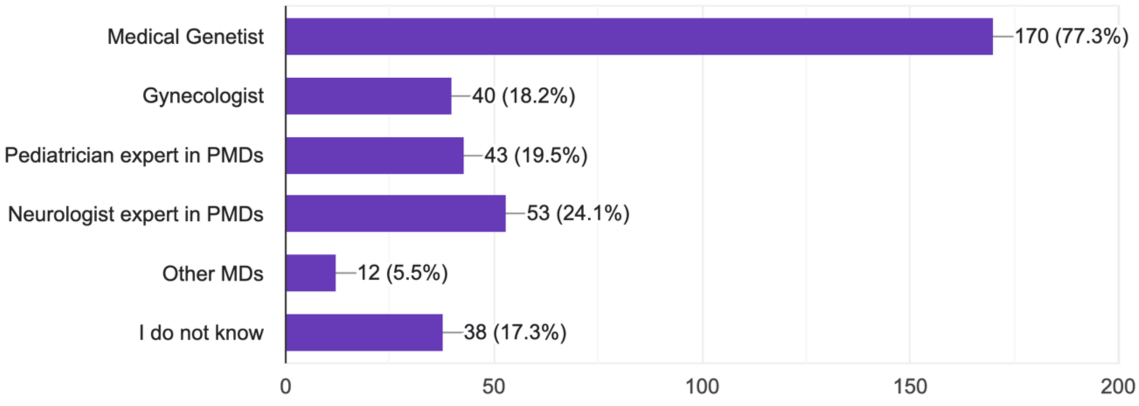

IS PGD AVAILABLE IN YOUR COUNTRY?

220 responses

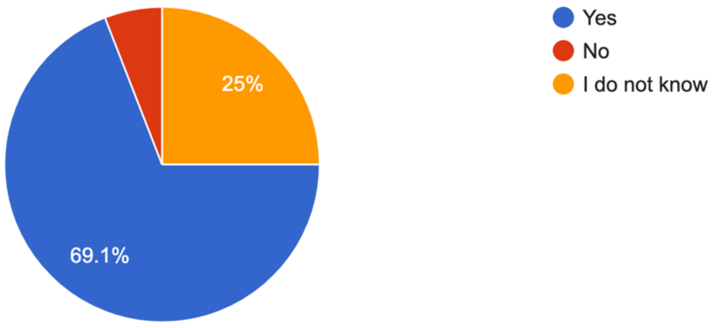

Is there a law of the management of PGD in your country?

220 responses

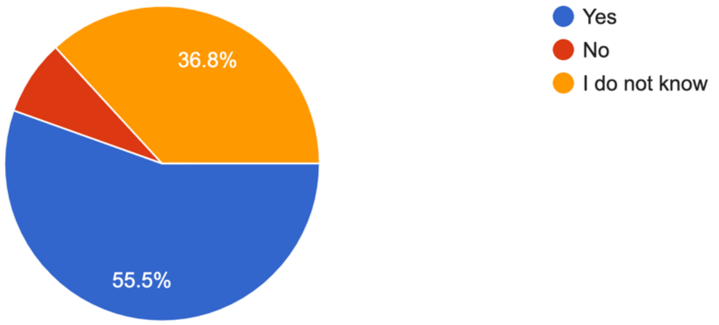

IS PGD PRECEDED BY A MEDICAL GENETIC COUNSELING CONSULTATION?
220 responses

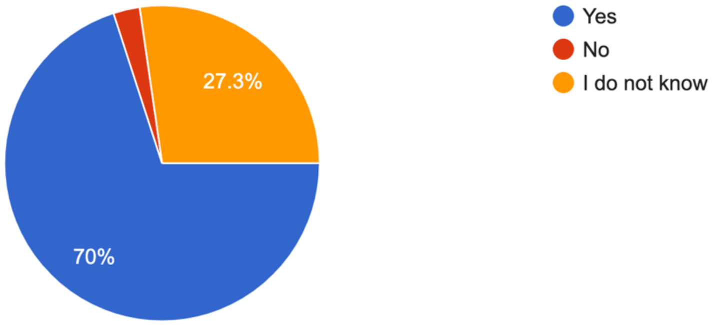

Do you prescribe the mitochondrial cocktail to your PMD patients
220 responses

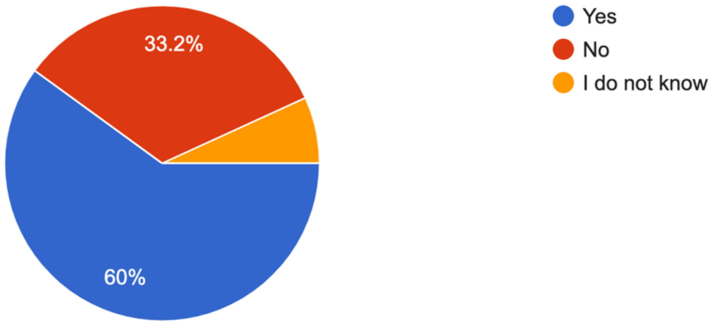

if you prescribe the mitochondrial cocktail, what do you usually consider (multiple choices allowed)
167 responses

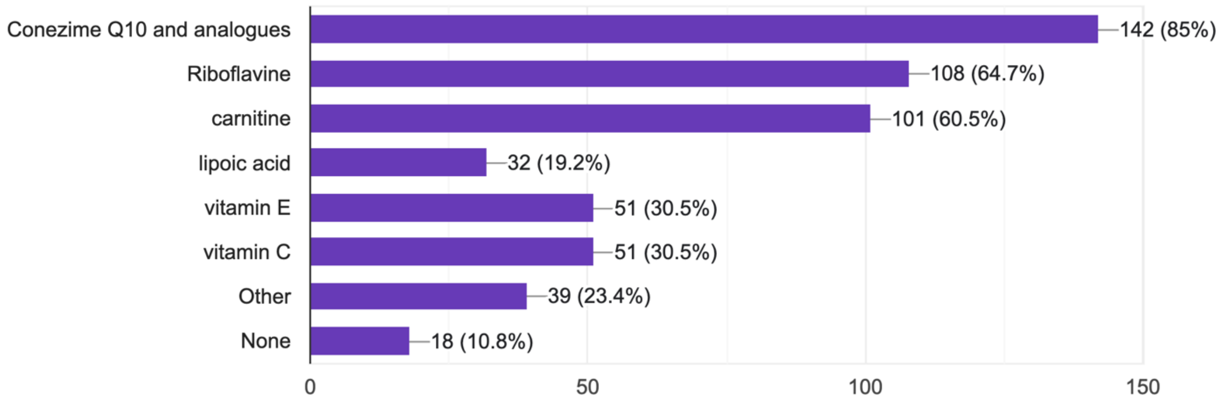

Is the mitochondrial cocktail reimbursed by the health system of your Country?

220 responses

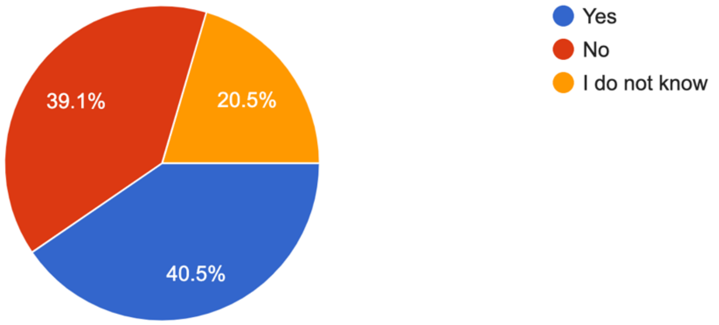

Is there a need for education and training in PMDs?

220 responses

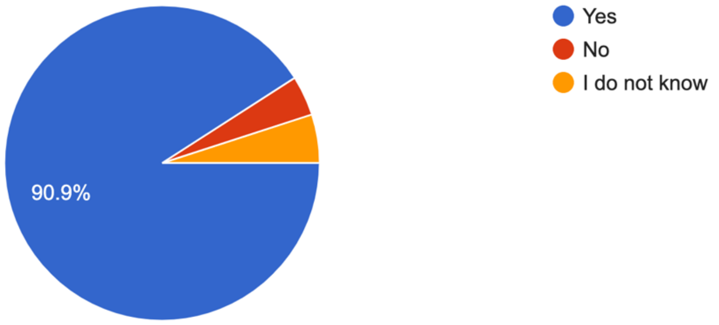

Is there a need for education and training in the residency programms in PMDs
220 responses

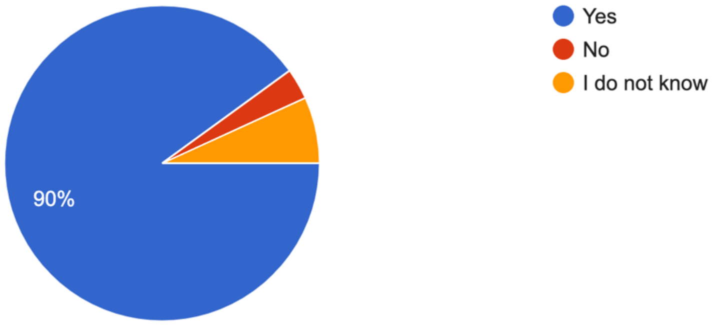

Do you think ERNs must provide more education on PMDs through different initiatives
220 responses

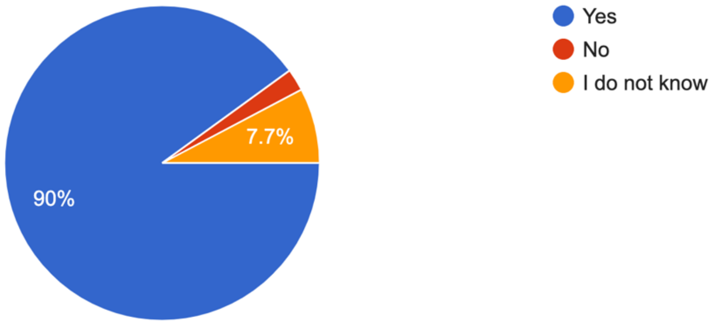

Supplement: Supplementary file 2 — The interERNs Survey results. (PDF 3234 KB) [file 415_2023_12017_MOESM2_ESM.pdf]
